# Complete Remission of Bone Metastases in Renal Cell Carcinoma with Nivolumab

**DOI:** 10.7759/cureus.5531

**Published:** 2019-08-30

**Authors:** Sowjanya Vuyyala, Shipra Gandhi, Joseph B Kuechle, Saby George

**Affiliations:** 1 Medicine, University Hospitals Seidman Cancer Center, Shaker Heights, USA; 2 Oncology, Roswell Park Cancer Institute, Buffalo, USA; 3 Orthopedic Oncology, Roswell Park Cancer Institute, Buffalo, USA

**Keywords:** renal cell carcinoma, bone metastasis, nivolumab, remission

## Abstract

A 60-year-old female, who presented with abdominal discomfort, was noted to have an enhancing left renal mass, with central necrosis on a CT scan. She underwent radical nephrectomy and biopsy revealed clear cell renal cell carcinoma, Fuhrman grade 4. After 1.5 years of her surgery, she developed metastatic disease with pulmonary nodules and was started on sunitinib. She had disease progression with development of a new 8.2 x 7.6 cm expansile, lytic bony lesion with a complete destruction of the left scapula and 5th left rib lesion. She was treated with Nivolumab for three years. Scans revealed complete resolution of the left scapular metastasis, left rib lesion and the pulmonary nodules. The patient experienced no skeletal-related event (SRE), and no bisphosphonates or receptor activator of nuclear factor-kappa B ligand (RANKL) inhibitor was used. The patient remains in complete remission, three years out of treatment. This case highlights the importance of exploring this particular class of drugs for renal cell carcinoma (RCC) with bone metastasis.

## Introduction

The estimated incidence for renal cell carcinoma (RCC) in the US is approximately 65,340 and 14,970 deaths are expected in 2018 [1]. Around 25-30% of the patients have distant metastatic or advanced loco regional disease at presentation and an additional 20-40% progress to develop metastatic disease after presenting with a localized disease [2]. Bone is the second most common site of metastases with 20-50% presenting with metastasis and about 20-35% of patients develop a skeletal lesion during the disease progression [3]. The most common sites of bone involvement are pelvis, ribs, spine followed by femur, humerus, and skull [4]. The lesions are osteolytic and add to significant morbidity. Treatment of the bone metastasis with check point inhibitors has currently not been extensively studied. We report a very interesting study with complete resolution of bone metastasis with nivolumab.

## Case presentation

**Figure 1:**
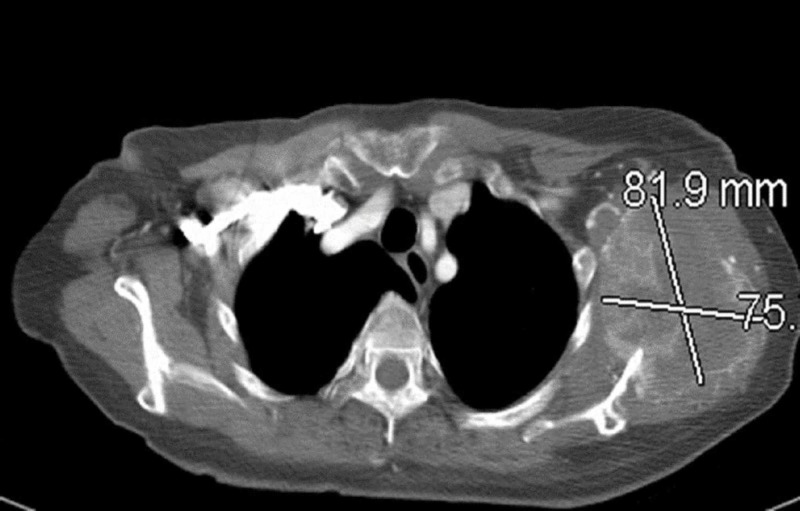
CT chest showing 8.2 cm x 7.6 cm expansile lytic lesion in the left scapula.

A 60-year-old Caucasian female with 10 pack-year of smoking presented with abdominal bloating in August 2009 and computed tomography (CT) of abdomen pelvis revealed a left renal mass measuring size 7.5 x 6.5 cm with central necrosis. She underwent laparoscopic left radical nephrectomy a month later, with pathology revealing a Fuhrman grade 4 clear cell carcinoma. The patient was monitored with serial CT of the chest and abdomen with no evidence of metastatic disease. A year and half later, she presented with generalized tonic-clonic seizures and was diagnosed with a solitary brain metastasis involving the left temporal lobe which was treated with gamma knife therapy. CT of the chest revealed new pulmonary nodules, 8.2 x 7.5 cm expansile lytic metastatic lesion with complete destruction of the left scapula and left 5th rib lytic lesion (Figure 1).

**Figure 2:**
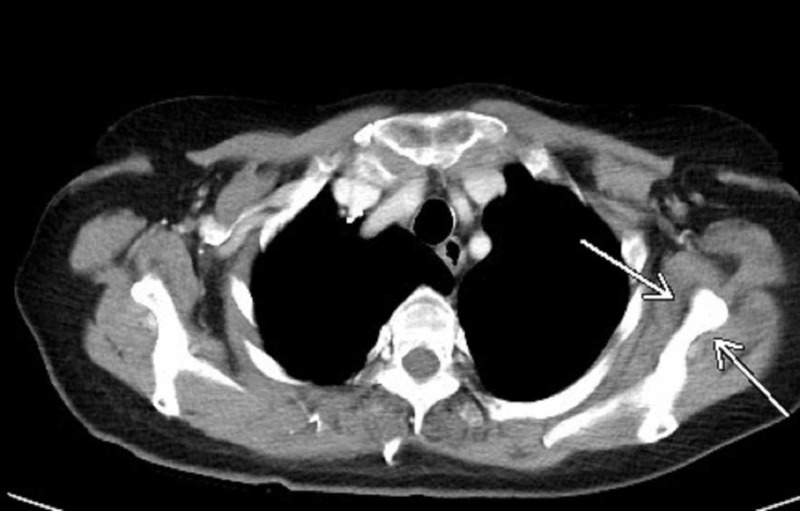
CT chest showing resolution of the lytic expansile scapular lesion, after three years of treatment with nivolumab.

The patient was treated with first-line anti-angiogenic agent sunitinib. However, due to disease progression noted six months later, she was started on a phase II clinical trial CA-209210, a randomized blinded dose-ranging study of BMS 936558, which later was named Nivolumab. There was continued regression of the metastatic lesions with complete resolution of bone and pulmonary metastasis noted over the next three years of treatment (Figure 2).

**Figure 3:**
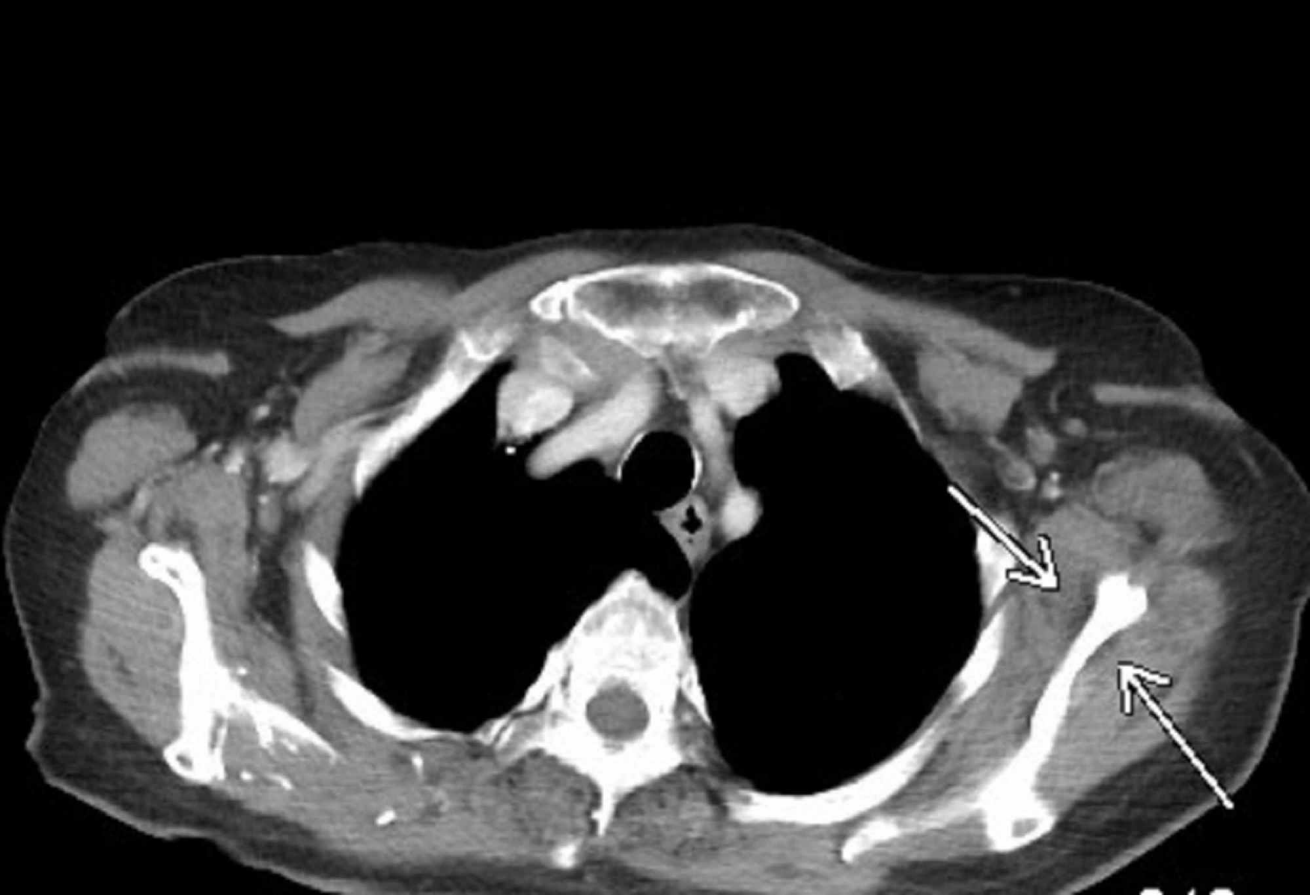
CT chest continues to show complete response of the osseous scapular lesion after being off nivolumab for three years.

The patient experienced no skeletal-related event (SRE), and she was not treated with bisphosphonates or RANK ligand inhibitors. She had discontinued from the clinical trial three years after initiating Nivolumab, due to burden of frequent appointments. It has been three years after the treatment was stopped, and the patient remains in complete remission (Figure 3).

## Discussion

Bone metastases (BM) from RCC are highly expansile, osteolytic and destructive with high rates of SREs. In a study by Wood and Brown, 80% of the patients with BM underwent radiotherapy to bone, 28% had surgery, 27% experienced spinal cord/nerve root compression and, 20% had pathologic fractures [5]. BM is a poor prognostic factor and is associated with shorter progression-free survival (PFS) and median overall survival after adjusting for other prognostic factors [6,7].

Bisphosphonates especially zoledronic acid and denosumab, a RANKL monoclonal antibody, have shown to significantly decrease the rate of SRE, improve overall survival, disease progression in patients with RCC [8,9]. Surgical interventions and radiation play a major role in the treatment of spinal cord compression, pathological fractures and reduce impending fractures [9].

The tumor cells create a conducive bone microenvironment by secreting signaling mediators like TGF-β that activate osteoblasts to increase the production of receptor activator of nuclear factor kappa B ligand (RANKL) which acts on the receptor on osteoclasts leading to its activation and bone resorption [10]. Disseminated tumor cells create “onco-niche” in the bone, evade immune surveillance, and are resistant to apoptosis. Programmed cell death ligand (PD L1) is overexpressed in up to 30% of RCC tumor cells which binds to PD 1 on the activated T cells leading to its down regulation and immune evasion. Nivolumab is human immunoglobulin - G4 immune checkpoint inhibitor antibody that blocks the interaction of PD 1 with ligands PD L1 and PD L2 and enhances the activity of T cell [11]. Randomized study CheckMate 025 has demonstrated significantly improved overall survival (OS) and objective response rate (ORR) with nivolumab compared to everolimus in advanced renal cell carcinoma. Subgroup analyses revealed improved median OS in patients with bone metastases treated with Nivolumab [12].

Our patient had presented with disease progression after initial presentation with a localized disease with metastases to brain, lung, and bone. She continued to have disease progression after six months of treatment with VEGF inhibitor sunitinib. She exhibited complete resolution of the lesions with three years of nivolumab and was able to tolerate the treatment with no adverse effects. She did not experience any SRE and is in complete remission, three years out of treatment. No bisphosphonate or RANKL inhibitor was used.

## Conclusions

To our knowledge, this is the first reported case of complete resolution of RCC bone metastases treated with nivolumab alone. This case highlights the efficacy of checkpoint inhibitors in the bone metastatic lesions by increasing the antitumor response and importance of exploring immune checkpoint inhibitors class of drugs for RCC with bone metastases. This suggests that the biology of disease that metastasizes to the bones is different and that may make them susceptible to checkpoint inhibitors like nivolumab. There is an unmet need to study the biology of this phenotype of RCC that preferentially metastasizes to bones. If future data continue to demonstrate significant response of bone metastasis to checkpoint inhibitors this may influence how aggressive with resection vs intralesional procedures we are for these patients.
